# QUANTITATIVE EVALUATION OF EXPERIMENTAL BONE REGENERATION USING INDENTATION TESTS

**DOI:** 10.1590/1413-785220172502164015

**Published:** 2017

**Authors:** Valéria Trombini Vidotto, Nilza Alzira Batista, José Ricardo Lenzi Mariolani, William Dias Belangero

**Affiliations:** 1 Universidade de Campinas (UNICAMP), Faculdade de Ciências Médicas, Laboratório de Biomateriais em Ortopedia, Campinas, SP, Brazil.

**Keywords:** Bone regeneration, Hardness tests, Animal experimentation

## Abstract

**Objectives:**

To determine whether the macroindentation test can be applied to quantitatively assess bone regeneration.

**Methods:**

A 3.2 mm diameter transverse monocortical defect was created on the medial aspect of both proximal metaphyses of the tibia of male Unib-WH rats. For the macroindentation tests, we used 5.00 mm diameter indenters with a 3.2 mm tip. Defect testing was performed 1 to 12 weeks following the surgical procedures to compare the hardness of the newly developed tissue over the 12-week study period. Additional histological, morphological and physical/chemical data were obtained by optical and electronic microscopy, Raman, and energy dispersive x-ray spectrometry (EDS).

**Results:**

The mean indentation forces increased in a time-dependent manner from 4 to 12 weeks (p<0.001). Tests performed with the 5.0 mm diameter tip were not able to measure the indentation forces in the first week after the procedure. Moreover, in the second postoperative week indentation forces and the newly formed tissue within the spinal canal were greater than those measured in the fourth and eighth weeks.

**Conclusions:**

The macroindentation test can be used to quantitatively assess bone regeneration in experimental studies. The choice of indenter tip diameter should consider the study design. ***Level of Evidence II, Diagnostic Studies.***

## RESUMO

**Objetivos:**

Determinar se o teste de macroendentação pode ser aplicado para avaliar quantitativamente a regeneração óssea.

**Métodos:**

Foi criado um defeito monocortical transversal com 3,2 mm de diâmetro na face medial de ambas as metáfises proximais da tíbia de ratos Wistar machos. Para os testes de macroendentação, empregou-se endentador com ponta de 3,2 mm e 5,0 mm de diâmetro. O teste dos defeitos foi realizado em 1 a 12 semanas depois dos procedimentos cirúrgicos, de modo que a dureza do tecido recém-formado foi comparada no período de 12 semanas do estudo. Os dados histológicos, morfológicos e físico-químicos adicionais foram obtidos por microscopia óptica e eletrônica, espectrometria Raman e EDS (espectrometria com dispersão de energia).

**Resultados:**

As forças médias de endentação aumentaram de modo dependente do tempo de 4 a 12 semanas

(p < 0,001). Os testes realizados com a ponta de 5 mm de diâmetro não foram capazes de medir as forças de endentação na primeira semana depois do procedimento. Além disso, na segunda semana, as forças de endentação e o tecido recém-formado no interior do canal medular foram superiores aos da quarta e oitava semanas.

**Conclusões:**

O teste de macroendentação pode ser utilizado em estudos experimentais para avaliar quantitativamente a regeneração óssea. A escolha do diâmetro da ponta do endentador deve considerar o desenho do estudo. Nível de Evidência II, Estudos Diagnósticos.

## INTRODUCTION

In 5-10% of fractures, bone healing takes longer than expected and bone consolidation is not achieved.^1^ This fact highlights the importance of developing novel therapeutic, clinical or surgical strategies to accelerate bone healing and avoid nonunion. Consequently, biomechanical tests to quantitatively assess bone repair are important tools to evaluate the efficiency of these strategies. Many biomechanical tests are used in animal models to evaluate implant performance, bone repair, and the quality of the newly formed bone, such as tensile, bending and torsion tests.^2^ ^-^ ^7^

The indentation test is used in mechanical engineering to determine the hardness of a material to deformation, and this test can also be used to quantify the hardness of newly developed tissue on bone surfaces. As the hardness of this new tissue gradually equals the hardness of intact bone, bone repair is likely to be successful. Micro and nanoindentation tests are commonly used for this purpose, but few studies can be found in the literature on the macroindentation test.^8^ ^-^ ^11^ To determine the efficacy of the macroindentation test in quantitatively assessing bone repair, we examined the 12-week follow-up of histological, morphological, and biomechanical findings in newly developed tissue in the tibias of rats which were subjected to monocortical perforation.

## MATERIALS AND METHODS

Because of the lack of knowledge about the statistical distribution of results which would be obtained in the tests, samples were initially chosen from four animals for each indentation test group and two animals for the histology/EDS/ERS/Raman groups. Preliminary processing of the results showed that some sample sizes needed to be increased, resulting in the use of one hundred and two male Unib: WH rats (*Rattus novergicus albinus,* Rodentia mammalia), 10(±2) weeks old, with a body mass of 350(±20) g. The animals were obtained from the Centro Multidisciplinar para Investigação Biológica na Área de Ciência em Animais de Laboratório (CEMIB) of the Universidade de Campinas (UNICAMP). All procedures were approved by the institutional review board (record 2497-1). The animals were randomly allocated in groups as described in Table 1.

**Table 1:** Allocation of the animals according to test groups.

Test		Follow-up time (weeks)						Total
		Control	1	2	4	8	12	
Indentation	3.2 mm indenter	4	5	7	6	4	4	30
	5.0 mm indenter	4	3	7	6	4	5	29
Histology	Transverse sections	2	2	3	3	2	2	14
	Longitudinal sections	2	3	3	3	2	2	15
EDS/ERS/Raman	-	2	2	3	3	2	2	14
Total								102

Following trichotomy and antisepsis of both hindlimbs, 88 animals were anesthetized via intravenous tail injection of ketamine

(70 mg/kg) and xylazine (5 mg/kg), and placed in a supine position on a surgical stand. Using an anterior longitudinal knee approach, the proximal anteromedial metaphysis of both tibias was exposed and a 3.2-mm transverse monocortical defect was created with a low-speed (130 rpm) electrical hand drilling machine. A 1.5-mm length stop device avoided over-penetration and perforation of the opposite cortical surface. Next, the skin was sutured with 3.0 nylon line. The animals were maintained in a plastic cage with wood shaving bedding, food pellets, and water at 25°C with 12h:12h light-dark cycles. They received Paracetamol solution (25 mg/kg) during the first 48 postoperative hours for analgesia. Full weight-bearing on the hindlimbs was immediately permitted. After a period of 1, 2, 4, 8, and 12 weeks, the animals were euthanized with a sodium pentobarbital overdose, and their tibias were harvested for biomechanical, histological, and morphological examination. Fourteen animals were not subjected to the surgical procedure and served as a control group. (Table 1)

The tibias were carefully placed on a molded bed made of plaster of Paris to maintain the surface of the defect parallel to the ground. The macroindentation test was performed using a EMIC universal testing machine. The indenter, which was attached to a 100 N load cell, was centered on the defect axis and positioned just above the surface of the newly formed tissue. Next, the indenter was lowered to a depth of 0.6 mm (t = 0.6 mm) at a speed of 0.1 mm/s. The tests were performed using either a 3.2-mm diameter or a 5.0-mm diameter spherical Brinell indenter. Curves for applied load vs. indenter penetration depth were plotted using test data which were continuously registered by the test machine software (TESC 3.04). The applied force for t = 0.5 mm (F_0.5_) of each curve was determined. Two-way analysis of variance (ANOVA) was used to compare the mean values of F_0.5_ between groups. Pairwise comparison was performed using the Scheffe test, and a significance level of 5% (α = 0.05) was considered.

The pair of tibias from 29 animals (a total of 58 tibias) was assigned to histological analysis. (Table 1) Samples were fixed (using 10% buffered formalin), decalcified (using tetrasodium EDTA), and stained with hematoxylin and eosin (HE) for optic microscopic examination (using a Leica DMLB device). Next, the microscopic images were captured (using a Leica DC 300 F device) and analyzed using IMAGE-PRO PLUS 4.5 software. Newly developed tissues were classified as granulation tissue, cartilaginous bone, newly formed bone, and bone.

The samples were then dehydrated (from 70% to 100% alcohol), embedded in polyester resin, and cut with a precision sectioning diamond saw (Bühler Isomet). The samples were excited with an argon laser (λ = 514.5 nm wavelength, ~6 mW), and their spectra were acquired using a Raman microscope (inVia model, Renishaw).

Scanning electron microscopy (SEM) was performed to study the topography of the newly developed tissue surface. Additionally, energy dispersive X-ray spectroscopy (EDS) (using a Zeiss EVO LS15 model coupled with an EDS system) was used to determine the chemical composition of the newly developed tissue within the defect.

## RESULTS

**Figure 1:**
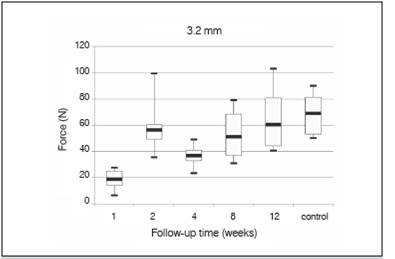
Penetration force achieved at 0.5 mm of a 3.2 mm indenter as a function of follow-up time.

**Figure 2:**
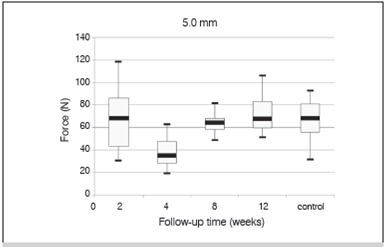
Penetration force achieved at 0.5 mm of a 5.0 mm indenter as a function of follow-up time.

Of the total 118 tibias (pairs of tibias from 59 animals), 4 were discarded due to fractures, and consequently 114 tibias underwent macroindentation testing. Indentation force increased in a time-dependent manner for both 3.2-mm and 5.0-mm diameter indenters. (Figures 1 and 2, and Table 2)

**Table 2:** Scheffe contrast between pairs of follow-up time means according to indenter diameter (α = 0.05, critical value = 2.519).

Indenter diameter (mm)	Follow-up times (weeks)	FS	Significant?
3.2	4 - 8	2.27	No
	4 - 12	3.80	Yes
	8 - 12	1.42	No
5.0	4 - 8	3.74	Yes
	4 - 12	5.14	Yes
	8 - 12	1.07	No

Nevertheless, data from samples taken from the 1-week follow-up could not be obtained with the 5.0-mm diameter indenter. Because the newly developed tissue was fragile, the indenter easily penetrated into the defect and reached its margins, invalidating the test.

**Figure 3:**
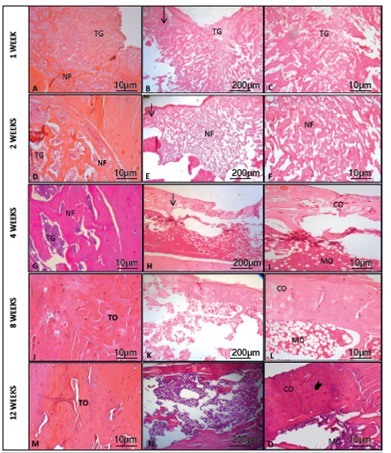
Transverse (A , D, G, J and M) and longitudinal sections (B, C, E, F, H, I, K, L, N and O) of the bone defect area after 1, 2, 4, 8 and 12 weeks (arrow: original-defect bone interface), with granulation tissue (GT), newly formed bone (NF), tissue in the cortical bone (CO) and medullar canal (MO), and osteocytes arranged concentrically around the central (Haversian) canals (lower arrowhead).

Histological examination revealed progressive primary bone regeneration within the monocortical tibial defect. At the 1-week follow-up we observed high-cellularity connective tissue composed predominantly of undifferentiated, spindle-shaped mesenchymal cells within the medullary canal. The initial stage of bone formation was also observed at the margins of the defect, where multiple ossification centers composed of osteoid (secreted by osteoblasts) and initial trabecular formation could be seen one week after tibial perforation. (Figure 3)

Two weeks after surgery, the defect site was filled with woven bone. Intense osteoblastic activity and dense connective tissue were observed within the intertrabecular space of the medullary canal. (Figure 3)

Four weeks after the procedure, a uniform pattern of osteogenesis could already be seen. Furthermore, the granulation tissue was completely resorbed, the woven bone was differentiated into cortical bone, and the medullary canal was remodeled. (Figure 3)

At 8 and 12 weeks post-procedure, the osteocytes reorganized into Haversian systems and complete remodeling of cortical and medullary bones was achieved. (Figure 3)

**Figure 4:**
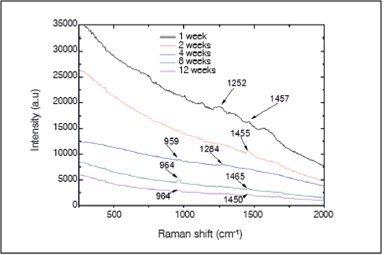
Raman spectra of the center of bone healing (cortical region of the defect). The bands indicate phosphate apatite (~960 cm-1), amide III (1200-1300

Raman spectra obtained from the surface of the cortical bone and medullary canal defects showed time-dependent decreases in phosphate apatite, amide III, and hydrocarbon (CH) side chain elements. (Figures 4 and ^5^) Images obtained with SEM are shown in Figures 6 to 10. In distinct areas of each sample, 4 to 6 points (center and margins of the defect and the medullary canal) were chosen to examine the chemical composition and mineral phase components using EDS. Table 3 indicates the proportion of calcium content at each point according to follow-up period.

**Figure 5:**
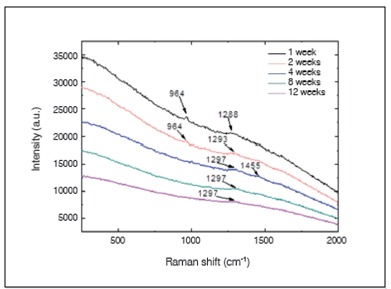
Raman spectra of the center of bone healing (medullar region). The bands indicate a) phosphate apatite (~960 cm-1), amide III (1200-1300 cm-1), and CH side chains (1450-1470 cm-1).

**Figure 6:**
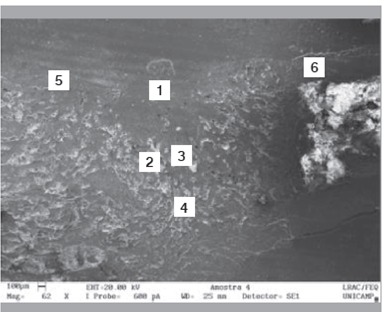
Longitudinal section of the defect at 1 week post-procedure.

**Figure 7:**
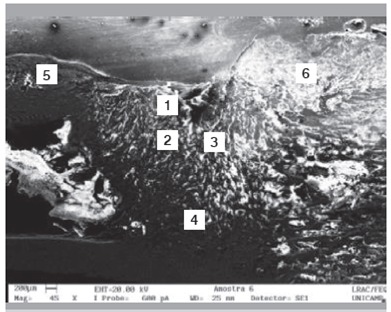
Longitudinal section of the defect at 2 weeks post-procedure.

**Figure 8:**
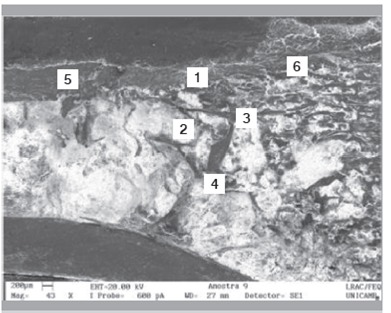
Longitudinal section of the defect at 4 weeks post-procedure.

**Figure 9:**
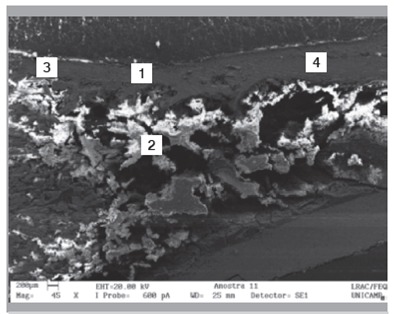
Longitudinal section of the defect at 8 weeks post-procedure.

**Figure 10:**
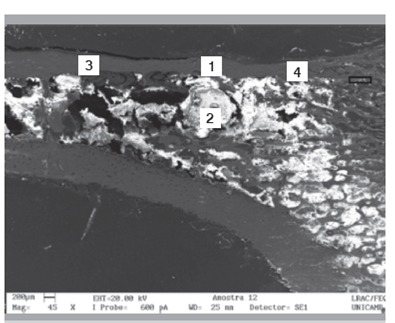
Longitudinal section of the defect at 12 weeks post-procedure.

**Table 3:** Calcium content obtained by EDS in different points of the samples according to follow-up time.

	Follow-up (weeks)				
Point	1	2	4	8	12
1	3.07	25.10	28.81	32,28	32.55
2	19.47	29.88	7.19	6.81	7.63
3	18.45	26.81	28.26	-	-
4	23.04	32.13	5.41	-	-
5	32.03	34.86	30.41	28.90	33.54
6	32.73	22.35	26.29	33.81	30.08

## DISCUSSION

Micro or nanoindentation tests are commonly used for mechanical evaluation of bone,^8^ ^-^ ^11^ but macroindentation testing is not common.^12^ This study describes a macroindentation test developed from the Brinell hardness test. Advantages of the Brinell hardness test include the fact that it can be performed using equipment which is easy to handle and its singular status as the only test used and accepted for materials with heterogeneous structural composition (such as bone).^13^

Instead of using the original method, we adapted the Brinell hardness test because it is difficult to determine the imprinted diameter on the bone, and it because a low magnitude force F would be required to avoid harming the newly formed and fragile tissue. Moreover, this force F would not allow the indenter to penetrate sufficiently, and would not yield reliable results.^14^

A 3.2 mm monocortical defect was chosen because it behaves like a bone fracture, which heals by primary intention when anatomically reduced and internally fixed.^15^ ^,^ ^16^ Furthermore, two diameters of indenter tips were evaluated (3.2 mm and 5.0 mm). Each indenter penetrated no more than 0.6 mm into the newly formed tissue. By penetrating 0.6 mm, the 3.2-mm diameter indenter produced an indentation with a diameter of 2.5 mm, and the 5.0-mm diameter indenter produced an indentation with a 3.2 mm diameter. Since the 5.0-mm diameter indenter covered a larger indentation area, it was more sensitive to positioning errors than the 3.2-mm diameter indenter. Table 2 confirms our expectation that from 4 to 12 weeks postoperatively the 5.0-mm diameter indenter applied greater force than the 3.2-mm diameter indenter. Since bone maturation occurs from margin to center, and the 5.0-mm diameter indenter reaches an area closer to the margin (i.e. resulting in greater strength at the margin than at the center of the defect), we expected the 5.0-mm diameter indenter to require greater force to penetrate 0.5 mm.

Also as expected, for most results F_0.5_ increased in a time-dependent manner. Surprisingly, samples obtained 2 weeks after the procedure revealed higher mean values than samples obtained 4 and 8 weeks later. (Figures 1 and 2) To explain those conflicting results, histological analyses were performed, and massive formation of tissue was seen within the medullary canal 2 weeks post-procedure. Furthermore, newly formed tissue remodeled at postoperative weeks 4 and 8. This leads us to hypothesize that one and two weeks after the procedure, massive tissue formation occurred in the medullary cavity and exerted a mass effect that resisted penetration by the indenter and consequently abnormally increased indentation force at 2 weeks post-procedure.

Because of its ability to effectively evaluate the presence of mineral (apatite phosphate) and organic components of bone's extracellular matrix (CH side chains and amide III), Raman spectroscopy is commonly used for structural assessment of bone healing and to provide information about the metabolic status of bone cells.^17^ ^-^ ^20^ ^)^ Here we found at the one- and two-week follow-ups that the band intensities of the cortical region spectra corresponded to the main collagen bands (i.e., amide III and CH side chains), while from the fourth to twelfth postoperative weeks, the band intensities of cortical region spectra corresponded to inorganic bone components (i.e., apatite phosphate); this indicated that mineralization of the newly formed tissue occurred between the second and fourth weeks after the procedure. Additionally, at 1 and 2 weeks following surgery, band intensities of medullary region spectra corresponded to apatite phosphate, while from the fourth to twelfth weeks after the procedure, the band intensities of the medullary region spectra did not correspond to apatite phosphate. These results are in accordance with biomechanical and histological findings, which showed time-dependent progressive increased indentation force and distinct healing processes observed in cortical and medullary bones, respectively, from 4 to 12 weeks post-procedure.

We also observed that the amount of collagen increased from center to the periphery of the defect. This finding was not a surprise since membranous bone ossification normally occurs from the periphery to the center of the defect. Four weeks post-procedure, the medullary canal was completely healed and exhibited a normal appearance, and the cortical bone was thicker and well formed.

In addition to chemical evaluation of bone repair, EDS of the defect site was performed. One week following surgery, the cortical region of the defect exhibited very low calcium content (3.07%) and no phosphorus; i.e., bone did not develop in that region. From the second postoperative week onward, the calcium content of the medullary canal decreased dramatically, reaching values from 5% to 8% after four weeks post-procedure. These results confirm the Raman spectroscopy findings. Our results also suggest that at the second postoperative week, a mass effect produced by isles of newly formed bone (composed mainly of hydroxyapatite) increased tissue resistance against indentation, while the cortical region of the defect was filled with immature bone (composed primarily of collagen). Subsequently, the newly formed tissue underwent progressive remodeling, so that four weeks after the procedure, the medullary canal was completely remodeled. As a result, the mass effect ceased and did not alter the hardness of the cortical bone, thereby permitting the indenter to penetrate with lower applied force.

Because the findings of this study indicate that macroindentation testing is adequate for biomechanical study of bone regeneration from four weeks post-procedure onward, two-way ANOVA and Scheffe tests were only applied to the results from this period (i.e., 4, 8, and 12 weeks post-procedure). Accordingly, macroindentation testing registered increased resistance in a time-dependent manner (p < 0.001).

The proposed model is more sensitive between four and eight weeks post-procedure. At eight weeks, the hardness of the defect was almost similar to the hardness of the control, but at 12 weeks, hardness of the defect was greater than that of the control. The Scheffe test showed a significant difference for the 5.0-mm diameter indenter between weeks 4 and 8 and between weeks 4 and 12. The 5.0-mm diameter indenter could detect bone maturation at 8 weeks post-procedure, but not from 8 to 12 weeks, most likely because there was complete cortical differentiation. Since the 3.2-mm diameter indenter could detect mechanical resistance in the newly developed tissue earlier than 5.0-mm diameter indenter, the 3.2-mm diameter indenter proved to be more adequate for macroindentation testing.

Bone repair from transverse monocortical defects occurs via intramembranous ossification. In contrast, bone repair from complete fractures occurs via endochondral ossification. Therefore, research on novel therapeutic methods for stimulating bone regeneration cannot be limited to the effect of intramembranous ossification. We believe that macroindentation testing should be used as a screening tool to identify therapeutic methods with the greatest potential to stimulate bone repair. Consequently, only therapeutic methods that exhibit positive results in macroindentation testing are worth testing in more complex experiment models such as osteotomy and osteosynthesis.

## CONCLUSION

Macroindentation testing of rat tibia defects is suitable for quantitative assessment of bone repair through evaluating the newly developed tissue hardness from 4 to 8 weeks following surgery. Chemical and histological examination corroborated the biomechanical results.
